# Acellular Tissue Engineered Vessels as Coronary Artery Bypass Grafts

**DOI:** 10.1016/j.jacbts.2025.101379

**Published:** 2025-09-11

**Authors:** Adam R. Williams, Kevin M. Nash, Robert D. Kirkton, Garyn S. Levitan, Melissa A. Daubert, Susan A. Whitney, Kaleb M. Naegeli, Abigail R. Benkert, Sharon L. McCartney, Heather L. Prichard, Laura E. Niklason, Alan P. Kypson

**Affiliations:** a Duke University Medical Center, Department of Surgery, Durham, North Carolina, USA; b Humacyte Global, Inc, Durham, North Carolina, USA; c Duke University Medical Center, Department of Medicine and Radiology, Durham, North Carolina, USA; d Duke University Medical Center, Department of Anesthesiology, Durham, North Carolina, USA; e University of North Carolina Healthcare, REX Cardiac Surgical Specialists, Raleigh, North Carolina, USA

**Keywords:** coronary artery bypass graft, coronary computed tomography angiography, regenerative medicine, spatial genomics, tissue engineering, vascular biology

## Abstract

Coronary artery bypass graft (CABG) uses the patient’s internal mammary artery and saphenous vein; however, unavailable or poor quality autologous vessels limit revascularization. This study addresses the critical need for alternative CABG conduits by evaluating a small diameter acellular tissue-engineered vessel ([sdATEV], 3.5 mm) in a primate model. Adult baboons (n = 5) underwent CABG to the right coronary artery (RCA) with an sdATEV. Patency, diameter, and cardiac function were longitudinally assessed by computed tomography angiography. All sdATEVs remained patent throughout the 6-month study. Computed tomography angiography demonstrated that the distal sdATEV diameter gradually remodeled to approximate the smaller baboon RCA. Histology and spatial transcriptomics revealed that sdATEVs recellularized with host endothelial and smooth muscle cells including a quiescent neomedia layer with gene expression patterns similar to the RCA, indicating that host cell ingrowth from the bypassed coronary artery regulates sdATEV diameter. These results suggest that the sdATEV is a durable alternative CABG conduit.

## Highlights

• Over the past 50 years, no novel CABG conduits have gained routine clinical use despite attempts with synthetics and xenografts.
• The sdATEV (3.5 mm) remained patent as an RCA CABG conduit through 6 months in a baboon surgical model.
• CTA revealed that adaptive host remodeling led to gradual tapering of the sdATEV to size-match the smaller baboon RCA.
• Quiescent host cells derived from the bypassed RCA media lined the sdATEV lumen and expressed vascular SMC and EC markers at 6 months.

## Visual Abstract

**Figure:**
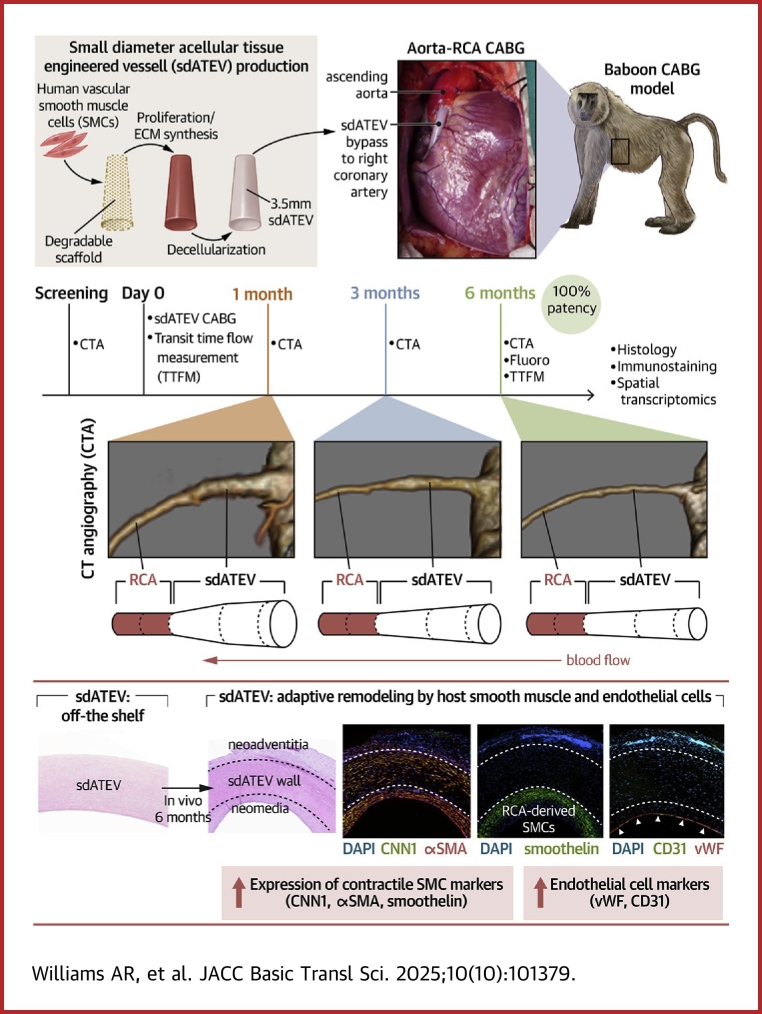

Coronary artery disease remains the leading cause of death worldwide. Despite advances in medical therapy and percutaneous coronary intervention, coronary artery bypass graft (CABG) is the standard of care for treating multivessel coronary artery disease.^1^^,^^2^ The left internal mammary artery is predominantly used to bypass the left anterior descending artery, whereas saphenous veins are commonly used for the remaining coronary targets. Arterial conduits such as autologous right internal mammary artery and radial artery have seen increased use following clinical trials demonstrating superior patency to saphenous vein, but their widespread adoption is still limited.^3^ Whereas saphenous vein grafts (SVGs) are used in 80%-90% of CABG cases,^4^ long-term patency is approximately 50% at 10 years.^5^ Furthermore, a substantial number of patients have veins that are of poor quality, or absent due to prior harvest or ablation, which limits complete coronary revascularization. Indeed, nearly 10% of patients enrolled in a recent large, multicenter trial were assigned to percutaneous coronary intervention instead of CABG, due to lack of usable vein or other conduit.^6^ In addition, 10%-20% of CABG patients require reoperation within 10 years due to prior graft failure,^7^^,^^8^ highlighting the critical need for alternative durable conduits.

Nonautologous vascular conduits have been used previously in CABG with limited success.^9^ Cryopreserved allogeneic SVGs have only 49% patency at 1 year,^10^ and xenograft conduits, such as bovine carotid arteries that are chemically treated to reduce antigenicity, have demonstrated poor durability with as low as 23% patency at 7 months following CABG.^11^^,^^12^ Small diameter (<5-mm) synthetic conduits, such as expanded polytetrafluoroethylene, are prone to thrombogenicity and are rarely used in CABG. Seeding the expanded polytetrafluoroethylene lumen with autologous endothelial cells (ECs) and culturing for several weeks provides some improvements in patency (95% patency at discharge, estimated 60% patency at 5 years),^13^ but this method is cumbersome and is not broadly applicable.

The acellular tissue-engineered vessel ([ATEV], Humacyte Global, Inc) is a bioengineered vascular conduit that is created from human vascular cells and then decellularized to yield a mechanically robust, off-the-shelf vessel composed of human extracellular matrix (ECM) proteins.^14^ ATEVs having 6-mm inner diameter and 40 cm of usable length have been evaluated in patients for the repair of traumatic vascular injuries,^15^^,^^16^ as conduits for hemodialysis access,^17^^,^^18^ and for treatment of peripheral artery disease.^19^^,^^20^ These studies have shown that, on implantation, the ATEV recellularizes with the patient’s cells to become a living blood vessel^18^ with long-term durability up to at least 6 years.^20^

Recently, we described production of a small diameter acellular tissue-engineered vessel ([sdATEV], 3.5-mm inner diameter) and its use as a subclavian-to-pulmonary artery Blalock-Taussig-Thomas shunt in a juvenile primate model.^21^ Here, we evaluate the feasibility, durability, and remodeling potential of the sdATEV over 6 months in an adult baboon CABG model.

## Methods

### Study design

This study aimed to evaluate the durability, patency, and biocompatibility of a small diameter tissue-engineered blood vessel as an alternative conduit for CABG in a 6-month nonhuman primate model. Adult male baboons (*Papio anubis*/*P cynocephalus*) weighing >30 kg were chosen for implantation given their phylogenetic similarity to humans, which obviates the need for immunosuppression. All procedures were approved by the Duke University Institutional Animal Care and Use Committee (protocol number A152-22-08) and were in compliance with the National Institutes of Health Guide for the Care and Use of Laboratory Animals. Animals were screened by preoperative angiography to select for baboons with right-dominant coronary anatomy and relatively larger proximal right coronary artery (RCA) diameter. A total of 5 animals were implanted with sdATEVs as aorta-RCA conduits (Supplemental Table 1).

### sdATEV production and characterization

**Figure 1:**
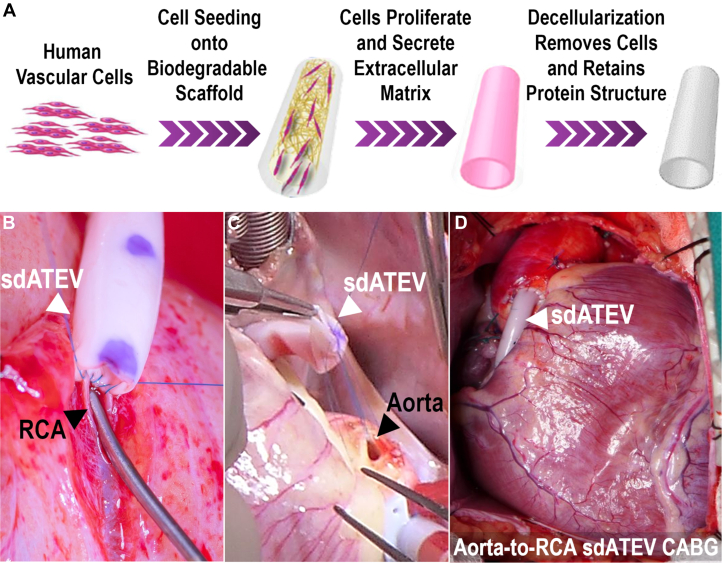
sdATEV Production and Implantation in a Baboon CABG Model (A) Schematic of small diameter (3.5-mm) acellular tissue-engineered vessel (sdATEV) manufacturing process. (B) Formation of distal anastomosis of 3.5-mm diameter sdATEV to native right coronary artery (RCA) in adult baboon showing size mismatch to ∼1.5-mm diameter RCA (1.5-mm stainless steel coronary probe is placed in RCA). (C) Proximal anastomosis of sdATEV to aorta using parachute suturing technique. (D) Final aorta-to-RCA configuration of sdATEV on the heart prior to closure. CABG = coronary artery bypass graft.

ATEVs having an internal diameter of 3.5 mm and length of 23 cm were produced using previously described methods^21^ (Figure 1A). Briefly, human vascular smooth muscle cells (SMCs) are seeded onto rapidly degradable polyglycolic acid tubular scaffolds secured within individual bioreactor bags. Over a culture period of approximately 8 weeks, cells proliferate and secrete human vascular ECM proteins, whereas the polyglycolic acid scaffold dissolves. In a final step, the engineered vessels are decellularized to remove antigenic cellular material, while retaining the human ECM and their mechanical properties.^14^ The sdATEV structural and mechanical properties were characterized previously.^21^ Values for wall thickness (489 ± 81 μm; n = 358), suture retention strength (217 ± 38 g; n = 361) and burst pressure strength (4887 ± 269 mm Hg; n = 76) were similar to, or exceeded, those of human internal mammary artery^22^^,^^23^ and saphenous vein.^22^, ^23^, ^24^ The sdATEV conduits can then be stored and used off-the-shelf for immediate use when indicated.

### Baboon CABG model

For the implantation, animals were sedated with ketamine (10-30 mg/kg) with or without dexmedetomidine (10-39 μg/kg) and anesthetized with inhaled isoflurane (0.5%-4%). A median sternotomy was performed, the heart was suspended in a pericardial cradle, and heparin (300 U/kg) was administered prior to initiation of central cardiopulmonary bypass. The sdATEV distal anastomosis to the RCA and proximal anastomosis to the ascending aorta were performed using a running 7-0 Prolene suture under cardioplegic arrest using cold Del Nido cardioplegia. Implanted graft lengths ranged from 2.0 to 3.5 cm. The proximal intervening RCA was ligated and divided to prevent competitive flow. Blood flow rate and pulsatility index in the sdATEV were measured intraoperatively by transit time flow measurement (MediStim VeriQ). Postoperatively, baboons received daily oral aspirin (10 mg/kg), clopidogrel (0.2-0.5 mg/kg), and pantoprazole (0.6-1.2 mg/kg). Animals were continuously monitored for postoperative complications and clinical signs of graft occlusion. Routine laboratory bloodwork was performed preoperatively, postoperatively, and at months 1, 3, and 6 after CABG. Animals were euthanized by KCl injection at 6 months and sdATEVs were harvested for histopathologic evaluation and spatial transcriptomics.

### CTA and fluoroscopic angiography

Animals were evaluated by coronary computed tomography angiography (CTA) at baseline (ie, prior to CABG), and at 1, 3, and 6 months postoperatively. Contrast (IsoVue 370) was administered intravenously, and retrospective ECG-gated acquisitions images were captured on a SOMATOM Force CT scanner (Siemens Medical Systems). Images were postprocessed using TeraRecon software to generate 3-dimensional reconstructions and to measure luminal diameters at the proximal, mid, and distal portions of the sdATEV graft and in the native RCA. The left ventricular ejection fraction and left ventricular chamber volumes were measured from CT images using end-diastolic and end-systolic phases and standard volumetric methods. Estimation of wall shear stress (WSS) was based on CTA-measured luminal diameters and average blood flow rates. Similar to the findings of Zilla et al,^25^ WSS was calculated as 4 μQ/πr^3^ where μ is blood viscosity, Q is volumetric flow rate, and r is vessel radius. Fluoroscopic angiography was performed on the first animal at 10 days and for all animals prior to explantation at 6 months. Following peripheral catheterization, the conduit was imaged by contrast injection (IsoVue 370) using an OEC 9800 Plus C-arm (GE Healthcare).

### sdATEV explant histology

Six months after implantation, a redo median sternotomy was performed and the sdATEV graft was exposed. Transit time flow measurement of the sdATEV was performed to quantify blood flow and pulsatility index. After euthanasia, the entire sdATEV with both anastomoses was explanted en bloc with pressure fixation at ∼120 mm Hg in 10% neutral buffered formalin. Explanted tissues samples underwent sectioning, dehydration, paraffin embedding, and subsequent sectioning (5-μm thickness) for histologic analysis. Hematoxylin and eosin and immunohistochemical staining were performed according to previously described methods^18^ (see Supplemental Table 2 for immunohistochemical antibody information). Brightfield hematoxylin and eosin images were captured using an Olympus BX41 microscope equipped with an Olympus DP25 camera and cellSens software. Fluorescence images of immunohistochemical staining were taken using a Nikon TE2000U microscope and a Photometrics IRIS 9 sCMOS camera. Image acquisition and processing were performed with μManager,^26^ NIS Elements (Nikon), or Fiji (ImageJ, National Institutes of Health) software.^27^

Cell proliferation was quantified using representative tissue cross-sections that were immunostained for Ki67. Imaged sections were divided into 4 quadrants, and further subdivided into sdATEV medial wall, neomedia ingrowth, and adventitia regions. The number of Ki67^+^ cells in each region was quantified as a subset of the total number of cells (identified by 4′,6-diamidino-2-phenylindole–staining of cell nuclei) in that region. The proliferation index was calculated from the ratio of Ki67^+^ cells to total 4′,6-diamidino-2-phenylindole–positive nuclei.

### Spatial transcriptomics

Midgraft sections (n = 3) of the sdATEV explanted from the 3 animals with low host reactivity and 1 section of native RCA (n = 1) were analyzed by spatial transcriptomics using the 10x Genomics Visium HD platform and the Visium Human Transcriptome Probe Set (version 2.0) following manufacturer’s recommended protocols (CG000685_RevA). The RCA sample was taken from one of the animals from which an sdATEV graft was also selected. Sequence homology of all human probe sequences to the *P anubis* genome was ascertained by BLAST (National Center for Biotechnology Information) prior to sample processing. Single 5-μm tissue sections of each sample were used for spatial transcriptomics. RNA-sequencing data of generated libraries were analyzed using Space Ranger (version 3.0.1) and Loupe Cell Browser (version 8.1.2), with all RNA-sequencing reads aligned to human genome build GRCh38-2020. Data were analyzed at binning resolutions from 8 to 32 μm (termed “spots”). Relative expression of individual genes were calculated from a median-normalized average count of detected RNA for each gene within the selected tissue regions. To normalize for differences in cellularity between sdATEV and RCA samples, median-normalized gene counts were then divided by the median-normalized count of ACTB messenger RNA in the corresponding tissue region. Intima, neomedia, media, and adventitia tissue regions were manually defined based on the patterns of computationally predicted spatial gene clusters overlayed on hematoxylin and eosin–stained tissue sections and used to determine the percentage of spots in a given region in which a gene of interest was detected.

### Statistical analysis

Data display and statistical analyses were performed using GraphPad Prism (GraphPad Software, Inc). Comparisons among more than 2 groups were performed using a 1-way analysis of variance, 2-way analysis of variance, or repeated measures mixed-effects analysis, as appropriate, with Dunnett post hoc test (comparisons to a control) or Tukey post hoc test (multiple pairwise comparisons) when data were normally distributed, or a Kruskal-Wallis test with Dunn post hoc test (multiple pairwise comparisons) when not normally distributed. Two group comparisons were performed using a 2-tailed paired (within group) or unpaired Student’s *t*-test (between group) when data were normally distributed or Mann-Whitney *U* test when not normally distributed. Significant difference was defined as a *P* < 0.05. All data are shown as mean ± SD. Normal distribution was assessed using the Shapiro-Wilk test.

## Results

### In vivo assessment of sdATEV CABG conduits

**Figure 2:**
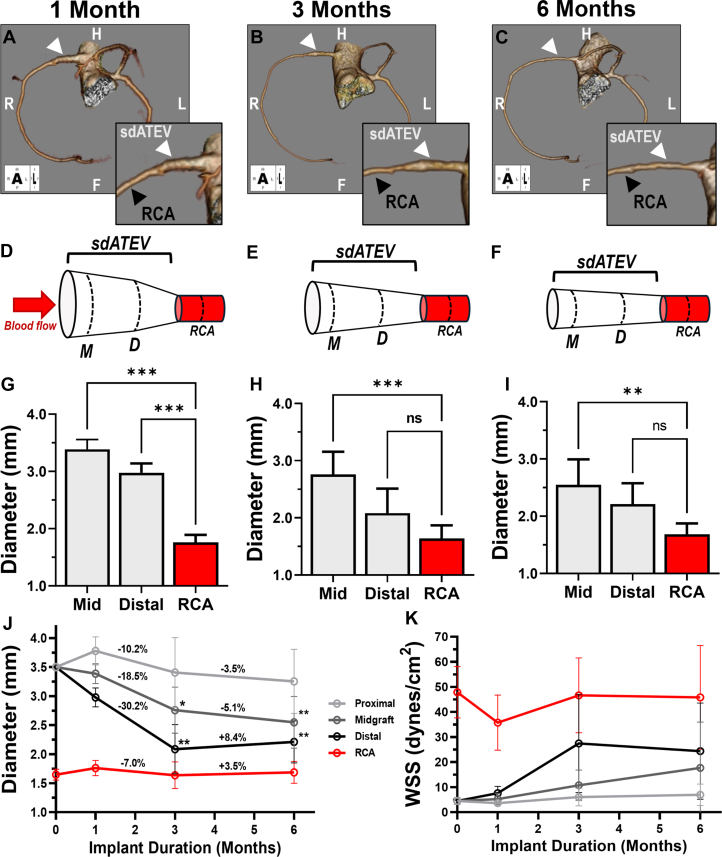
sdATEV Luminal Remodeling Followed In Vivo by 3D CTA (A to C) Representative 3-dimensional (3D) computed tomography angiograms from 1 to 6 months showing luminal remodeling to mimic RCA diameter. White arrows indicate sdATEV. (D to F) Diagrammatic representation of luminal remodeling from 1 to 6 months. (G to I) Quantification of luminal diameter from 1 to 6 months across 5 animals; mean ± SD; ∗∗*P* < 0.010; ∗∗∗*P* < 0.001; 1-way analysis of variance with Dunnett post hoc test vs RCA. (J) Changes in luminal diameter (percentage of change labeled from 1 to 3 and 3 to 6 months), Significance (∗*P* < 0.050 and ∗∗*P* < 0.010) only found in comparison with respective 1-month measurements, all other comparisons not significant; 2-way analysis of variance with Tukey post hoc test. (K) Calculated wall shear stress (WSS) of RCA and sdATEV during study; mean ± SD. CTA = computed tomography angiography; D = distal; F = foot; H = head; L = left; M = midgraft; NS = nonsignificant; R = right.

The sdATEV was evaluated in 5 baboons determined preoperatively to have a right-dominant coronary system (Figures 1B to 1D, Video 1). All animals reached the 6-month study endpoint without serious adverse events or evidence of clinical dysfunction of the sdATEV. Pos-operative changes in bloodwork were transient, and all values returned to baseline levels at 1 month. Physiologic function of the grafts was assessed with ultrasound transit time flow measurement. The average blood flow rate through the sdATEV was 32.4 ± 10.3 mL/min at implantation, with a pulsatility index of 1.7 ± 0.9. At 6 months, average blood flow was 35.0 ± 13.3 mL/min and the pulsatility index was 1.2 ± 0.0. These blood flow rates are similar to those reported for an adult baboon coronary artery (29.5 ± 14.0 mL/min).^25^ Furthermore, CTA demonstrated that all sdATEV conduits remained patent from 1 to 6 months postoperatively without focal stenosis or thrombosis. Representative images are shown in Figures 2A to 2C; CTA images for all animals are shown in Supplemental Figure 1. Fluoroscopic angiography was performed at an early time point for 1 animal (Video 2) and for all animals at the 6-month explantation (Video 3, Supplemental Figure 2). There was no aneurysmal dilatation, pseudoaneurysm, or mechanical breakdown of any of the sdATEVs at any time point.

At the time of implantation, the 3.5-mm sdATEV luminal diameter was roughly double that of the native RCA diameter (1.65 ± 0.09 mm preoperatively in 3 animals). During the following 6 months, the diameter of the RCA remained unchanged, whereas the lumen of the sdATEV gradually decreased to more closely resemble that of the native RCA (Figures 2D to 2J). Luminal diameter narrowing of the sdATEV was not confined to the anastomotic regions, but rather gradual tapering was seen throughout the length of the sdATEV, from 3.5-mm initial diameter at implantation to 1.7-2.6 mm at 6 months. The amount of luminal remodeling correlated with proximity to the RCA anastomosis. The average percentage of reduction in sdATEV luminal diameter was greatest from 1 to 3 months after implantation, predominantly affecting the mid and distal sdATEV regions, and then remained relatively unchanged from months 3 to 6 (Figure 2J). At 3 and 6 months, measured diameters of the distal sdATEV were not statistically different from those of the native RCA (Figures 2H and 2I). In no instance was the diameter of the sdATEV narrower than that of the corresponding RCA to which it was anastomosed.

As shown in Figure 2K, the calculated WSS of the native RCA was similar to that previously reported in the baboon left anterior descending artery^25^ and did not significantly change during the 6-month study (average 42.2 ± 13.9 dynes/cm^2^). At 1-month post-implantation, average calculated WSS through the proximal, middle, and distal portions of the sdATEV was 3.7 ± 1.2, 5.4 ± 2.4, and 7.9 ± 3.0 dynes/cm^2^, respectively, which was much lower than that calculated for native RCA. From 1 to 6 months, there was a ∼250% increase in calculated WSS within the distal portion of the sdATEV, that stabilized around 22.9 ± 16.6 dynes/cm^2^. The final calculated WSS in the distal sdATEV at 6 months was not significantly different from the WSS in the adjacent RCA (*P* = 0.083, 1-way analysis of variance with Tukey post hoc test). These results suggest that low WSS in the sdATEV near the distal anastomosis stimulated progressive vessel remodeling that consequently increased WSS within the sdATEV. Stabilization of the luminal diameter then occurred after the 3-month measurement, when distal sdATEV diameter and WSS were closer to that of the native RCA.

### Cardiac function after CABG

Postoperative CTA showed that end-diastolic volume, end-systolic volume, and stroke volume remained similar to preoperative levels throughout the 6-month study (Supplemental Figure 3A). Preimplantation left ventricular ejection fraction was 64.3 ± 7.1%, which did not change significantly at 1, 3, or 6 months (65.7% ± 6.1%, 64.1% ± 5.8%, and 62.6% ± 8.2%; *P* = 0.92) (Supplemental Figure 3B). There were no regional wall motion abnormalities detected, and all animals maintained preserved cardiac contractility at all time points.

### Histologic evaluation of sdATEV explants

**Figure 3:**
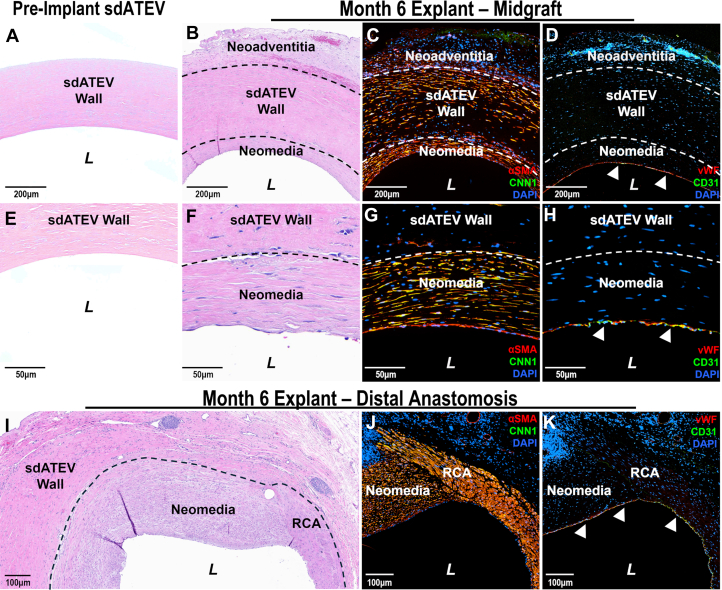
sdATEV CABG Repopulation With Vascular SMCs and ECs (A and E) Hematoxylin and eosin (H&E)-stained sdATEV preimplantation shows extracellular matrix proteins but no nuclei (lack of hematoxylin, blue). (B and F) H&E stain of midgraft explanted at 6 months shows remodeling into multilayered tissue with surrounding adventitia and luminal neomedia tissue. (C and G) α-Smooth muscle actin (αSMA, red) and CNN1 (green) identifies smooth muscle cells (SMCs) in sdATEV wall and neomedia. (D, H) VWF (red) and CD31 (PECAM1, green) show endothelialization of sdATEV lumen (white arrows). Cell nuclei were stained blue with 4',6-diamidino-2-phenylindole (DAPI). (I) H&E stain of sdATEV-RCA anastomosis at 6 months shows neomedia originating from RCA. (J) Neomedia contains cells expressing αSMA (red) and CNN1 (green). (K) Endothelial cells (ECs) expressing VWF (red) and CD31 (green) line the lumen of both the RCA and sdATEV neomedia across the anastomotic interface (white arrows). L = lumen; other abbreviations as in Figure 1.

At the 6-month explantation (Video 4), sdATEV histopathology showed that the previously acellular conduit (preimplant) (Figures 3A to 3E) was remodeled into a multilayered tissue without evidence of significant inflammation, thrombosis, or foreign body reaction (Figure 3B). Recellularization of the sdATEV by host cells was noted in all sections studied. Serial cross sections showed that the sdATEV wall was repopulated with cells that stained positive for α-smooth muscle actin (αSMA) and CNN1 (Figure 3C). αSMA and CNN1 coexpression is indicative of contractile SMCs.

Histology of the sdATEV explants did not demonstrate “neointima” of the classic form seen in vein and arterial grafts. Rather, there was an inner cellular layer lining the lumen of the sdATEVs containing αSMA^+^ and CNN1^+^ cells (Figure 3G). Based on histologic sections at the anastomoses, this tissue layer was derived from transanastomotic ingrowth of medial cells from the adjacent coronary artery (Figure 3I to 3K). This tissue ingrowth is therefore defined as “neomedia.” Luminal cells lining the neomedia expressed VWF and CD31 (PECAM1), indicative of an EC phenotype (Figures 3D and 3H). The source of the luminal endothelium could be transanastomotic from adjacent native vasculature (Figure 3K), similar to the neomedia, or from circulating progenitor cells.

### Measurement of cellular proliferation at month 6

**Figure 4:**
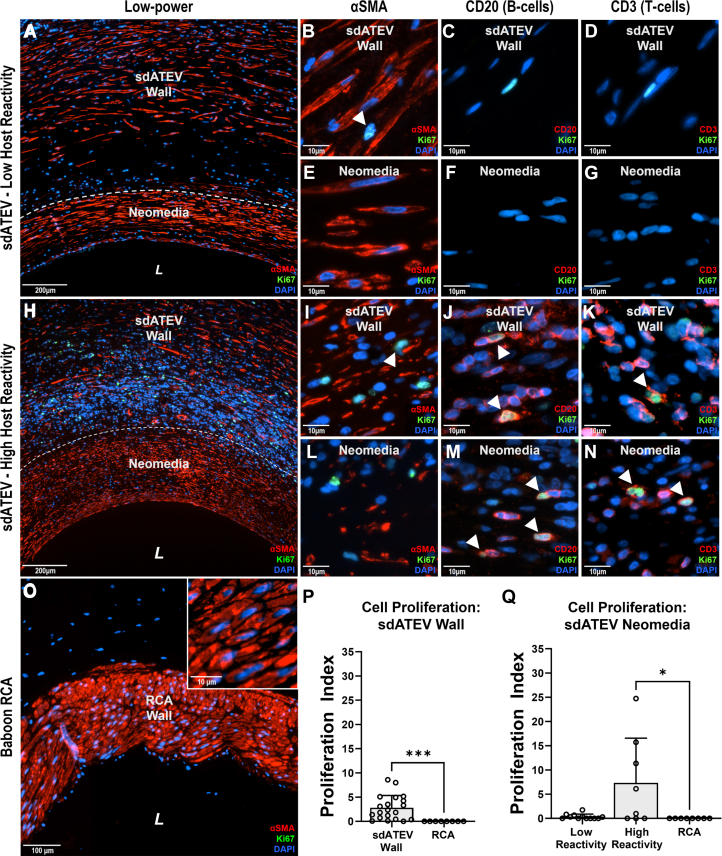
Low Cell Proliferation in sdATEV Wall and Neomedia at 6 Months (A to G) Six-month sdATEV explants from baboons with low host reactivity (n = 3) had few Ki67^+^ cells coexpressing αSMA (white arrows show coexpressing cells), and none coexpressing CD3, or CD20 with negligible Ki67^+^ cells in the neomedia. (H to N) sdATEV explants from baboons with high host reactivity (n = 2) exhibited more Ki67^+^ cells, predominantly CD20^+^ B cells and CD3^+^ T cells in both the sdATEV wall and neomedia. (O) No Ki67^+^ cells within the baboon native RCA. (P) Cellular proliferation in the sdATEV wall (2.8%) was higher than native RCA (0%, *P* < 0.010). (Q) Highly reactive animals had greater neomedia cellular proliferation (*P* < 0.050) than that of low-reactivity animals and native RCA, which were not significantly different. Mean ± SD; ∗*P* < 0.050; ∗∗*P* < 0.010; Kruskal-Wallis test with Dunn post hoc test. Abbreviations as in Figures 1 and 3.

We assessed whether repopulating SMCs in the sdATEV vessel wall and neomedia were quiescent or rapidly dividing at the 6-month time point using the nuclear proliferation marker Ki67 (Figures 4A to 4Q). The cells in the walls of the 5 sdATEV midgraft explants displayed a low level of proliferation (2.8% ± 2.6%) (Figure 4P). Consistent with ongoing cellular repopulation of the sdATEV wall, proliferating cells coexpressed Ki67 and αSMA (Figure 4B). In contrast, cells within the neomedia in 3 of 5 explant tissues displayed an extremely low proliferation index of 0.35% ± 0.52% (Figure 4Q). The low proliferation rate was similar to that of control samples of native baboon RCA, which showed no Ki67^+^ cells in the arterial wall (Figure 4O). However, neomedia in 2 of the explants contained cells with a proliferation index of 7.4% ± 9.2% (Figure 4Q).

Costaining of Ki67 with αSMA was not observed in the neomedia for any animal, implying that the proliferating neomedia cell population was not vascular SMCs. In contrast, colocalization of Ki67 with both CD3 (T lymphocytes) and CD20 (B lymphocytes) was noted in the neomedia of the 2 explants having higher cell proliferation rates (Figures 4M and 4N), implying that xenoreactive lymphocyte populations were proliferating in these 2 animals.

### Local signals supporting SMC quiescence

**Figure 5:**
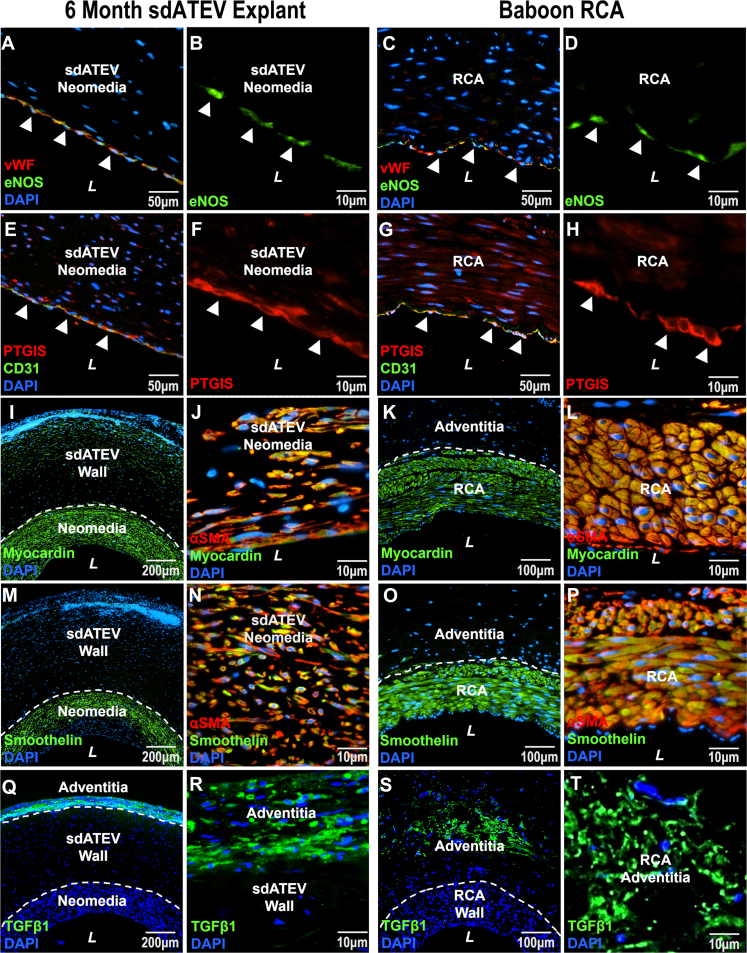
SMC and EC Protein Expression Indicative of Quiescent sdATEV Neomedia Representative immunohistochemistry staining of 6-month sdATEV midgraft explants (left 2 columns) and RCA (right columns). (A to D) ECs express both VWF and endothelial nitric oxide synthase (eNOS) on the lumen of the sdATEV and RCA. (B to H) Prostacyclin PGI_2_ synthase (PTGIS) is expressed by CD31^+^ luminal ECs as well as some CD31^−^ cells within the wall of both the sdATEV and RCA. (I to L) Expression of contractile SMC marker myocardin was colocalized with αSMA in the neomedia and sdATEV wall, whereas smoothelin (M-P) was colocalized with αSMA predominantly in the neomedia and not the sdATEV wall. Both myocardin and smoothelin colocalized with αSMA in the RCA, which matches the sdATEV neomedia and indicates its origination from the RCA media. (Q to T) TGFβ1, which may be associated with synthetic SMCs, was only found in the sdATEV and RCA adventitia. Abbreviations as in Figures 1 and 3.

To evaluate the role of ECs in regulating SMC quiescence in sdATEV explants, we immunostained for endothelial nitric oxide synthase (eNOS), constitutive prostacyclin PGI_2_ synthase (PTGIS), and endothelin-1 (ET-1) within the explanted sdATEV and RCA tissue. Endothelial expression of eNOS and PTGIS could decrease proliferation of local SMCs, whereas expression of ET-1 might increase smooth muscle proliferation. ECs expressing CD31^+^ and VWF^+^ on the lumen of both the sdATEV and the native RCA exhibited strong signal for both eNOS and PTGIS (Figures 5A to 5H). PTGIS expression was also weakly present in cells within the neomedia layer and sdATEV wall (Supplemental Figures 4A and 4B). These observations support a possible role for EC expression of factors restraining smooth muscle proliferation. In contrast, ET-1 staining was rarely observed in luminal cells of either sdATEV or RCA explants (Supplemental Figures 4C and 4D). Cells within the sdATEV neomedia layer predominantly expressed the contractile SMC markers myocardin and smoothelin, whereas transforming growth factor-β (TGFβ1) was localized in cells within the adventitia, similar to what was observed in native RCA (Figures 5I to 5T). Smoothelin expression was higher in the neomedia than the sdATEV wall suggesting that cells within the neomedia originated from the native RCA.

### Spatial transcriptomics of RCA and sdATEV explants

**Figure 6:**
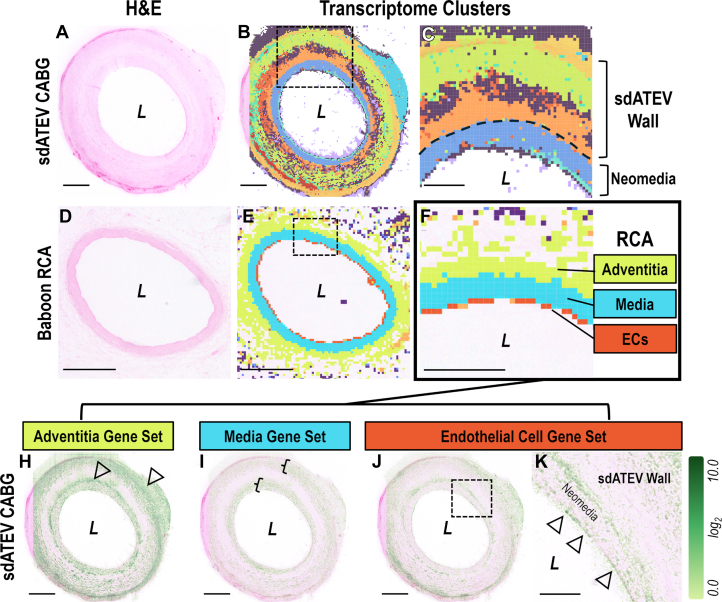
Spatial Transcriptomics Reveals Similarity of Explanted sdATEV to Native RCA Cross-sectional images and gene expression profiles of sdATEV midgraft (A to C) and native RCA (D to F) from the same animal. H&E stained (A, D) images of tissue sections analyzed by spatial transcriptomics with overlays of computationally predicted clusters (B, E) at 32-μm resolution. (Clusters colored independently between B to C and E to F). (H to K) Log-normalized sum of gene expression within the sdATEV for gene sets derived from RCA clusters at 8-μm resolution. (H) Adventitia genes localize primarily to the outer sdATEV wall and neomedia interface (arrowheads). (I) Media genes primarily expressed in neomedia and outer sdATEV wall (brackets). (J to K) EC genes expressed along lumen (K, arrowheads) and at neomedia interface. Bars = 1 mm for low magnification and 200 μm for high magnification. Gene sets listed in Supplemental Table 4. Abbreviations as in Figures 1 and 3.

High definition spatial transcriptomics libraries^28^ were generated from tissue sections of sdATEV (n = 3) and native RCA (n = 2) explanted at 6 months. Whereas this approach used a human messenger RNA probe library, 91.8% of human probe sequences had homology to 1 or more *P anubis* sequences, indicating a high degree of sequence conservation and accurate gene detection for *P anubis* genes. The spatial resolution of this transcriptomics approach allowed for analysis of contiguous gene expression across tissues in subdivisions from 8 to 32 μm, with lower resolution assisting in the identification of highly localized and low abundance transcripts. These subdivisions (“spots”) correspond to nearly single-cell resolution within the tissue. In all samples, unsupervised clustering of spots produced clusters corresponding to the layered organization of the sdATEV graft or native baboon RCA (Figures 6A to 6F).

To assess the genetic similarity of the repopulated sdATEV to the native RCA, we identified the 50 most differentially expressed genes from each computationally generated layer of the RCA at 32-μm resolution and calculated the average expression of these gene sets in the sdATEV graft from the same animal (Figures 6H to 6K). The most expressed genes of the RCA layers that contribute to gene expression patterns in the sdATEV grafts are shown in Supplemental Table 3. Adventitia genes are expressed highly at the outer edge of the sdATEV wall and the interface between the sdATEV wall and the neomedia. Genes distinguishing the media of the RCA media also colocalize to the sdATEV wall and neomedia showing a high degree of relatedness between these tissues. RCA endothelial genes colocalize along the sdATEV lumen and at the interface of the sdATEV wall and neomedia. Whereas the localization of prominent adventitial, media, and endothelial gene expression from the RCA correspond to respective localizations in the sdATEV graft, their expression patterns are more mixed throughout the sdATEV wall, implying that the repopulated sdATEV had a less organized structure at this 6-month time point than mature RCA.

**Figure 7:**
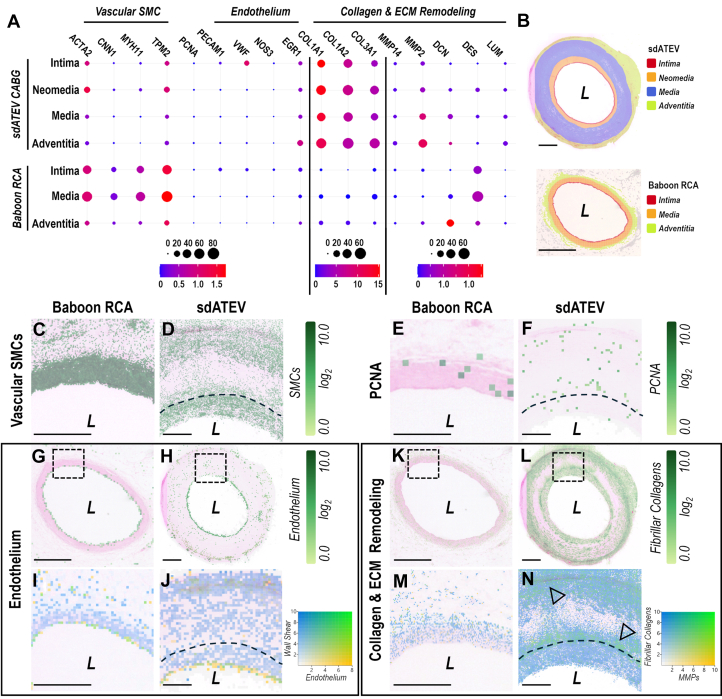
Comparative Gene Expression in Explanted sdATEV and Native RCA (A) Spatial transcriptomic gene expression patterns within 6-month sdATEV and RCA. Dot size indicates percentage of 8-μm resolution transcriptomic spots expressing the gene, coloration represents expression relative to *ACTB*. (B) Computationally predicted gene clusters overlayed on H&E-stained sections to define tissue regions in A. (C-D) Expression of vascular SMC genes (8-μm resolution) and (E to F) *PCNA* (32-μm resolution). (G to J) EC gene expression (32-μm resolution) with insets (black boxes, I-J) depicting coexpression (green) of EC genes (yellow) with endothelium shear stress genes (blue). (K-N) Expression of fibrillar collagens (8-μm resolution) with insets (black boxes, M to N) illustrating co-expression (arrowheads, green) of fibrillar collagens (blue) with matrix metalloproteases (yellow). Scale plots (log_2)_ represent sum of gene expressions in the selected family. Bars = 1 mm, low magnification; 200 μm, high magnification. Gene sets listed in Supplemental Table 4. Abbreviations as in Figures 1 and 3.

Spatial transcriptomics further probed the relative expression magnitude and distribution of specific vascular cell genes (Supplemental Table 4) within the RCA and sdATEV tissue layers (Figure 7A). Whereas the cell-dense RCA had overall higher relative gene expression than the actively remodeling sdATEV, there were analogous intensities of expression of SMC and EC markers, indicating a high degree of cellular similarity in these 2 tissues. Fibrillar collagens and ECM remodeling genes including matrix metalloproteases, *DCN*, and *LUM* were more abundant in cells within the sdATEV than the RCA, which indicates an active remodeling process in the graft tissue.

Contractile SMC gene expression was evident throughout the tissue layers (Figures 7B and 7C, Supplemental Figure 5) with very low expression of *PCNA* in the sdATEV wall and neomedia (Figures 7A, 7D, and 7E), which agrees with the low number of Ki67^+^ cells identified by immunostaining (Figure 4). Genes typically expressed in ECs were found within a thin layer on the luminal side of the RCA and sdATEV graft (Figures 7J to 7K). Additionally, expression of the transcription factor *EGR1*, a marker of WSS,^29^ was present within this layer in both the native RCA and sdATEV (Figures 7H and 7I), suggesting the presence of functional endothelium that is responsive to blood flow. The sdATEV also displayed regions of host remodeling “hotspots” defined by colocalization of matrix metalloproteases and fibrillar collagens (Figures 7J to 7M).

## Discussion

This study is the first to describe the use of a tissue-engineered blood vessel in a nonhuman primate model of CABG. The sdATEV is an off-the-shelf, acellular vascular conduit produced from human allogeneic donor cells that are removed at the final step of manufacturing to yield a readily available vascular conduit that could be used by surgeons without the need for graft harvest. The phylogenetic similarity of baboons to humans allowed long-term implantation of the human ECM-containing ATEV, without immunosuppression.^14^ Additionally, despite the size mismatch between proximal epicardial vessels in the baboon (∼1.7 mm) and adult human coronaries (2.8-3.9 mm),^30^ baboons are large enough to permit surgical implantation of an adult human–sized vascular conduit in an aortocoronary configuration, and have previously been used to study vein grafts.^25^^,^^31^ Blood flow rates, patency, and cardiac function were maintained in all 5 animals for the duration of our study, illustrating the physiological function of the 3.5-mm sdATEV in this challenging surgical model. The grafted territory remained perfused in each animal, and there were no impairments to cardiac function over the duration of the study. The maintenance of blood flow rate from implantation to 6 months supports the lack of any evolving, hemodynamically significant luminal narrowing, despite remodeling of the lumen by RCA-derived neomedia tissue. Measured pulsatility indices were below 2.0 throughout the experiment, which is consistent with adequate conduit blood flow rates in CABG.^32^

Criteria for a successful novel CABG conduit are rigorous: small luminal diameter must be combined with durable mechanical properties and a resistance to thrombosis. Importantly, the conduit should be free from allogeneic or xenogeneic immune reactivity, which can decrease conduit patency and function. Perhaps because of these criteria, over the past 50 years there have been no novel conduits for CABG that have entered into routine clinical use. Previous attempts to use fixed animal xenografts,^33^ synthetic polymers with,^13^ or without,^34^ ECs, and human cryopreserved vein have not proven successful. Recently, novel electrospinning methods have been used to create a biodegradable “biorestorative polymer” conduit with an embedded nondegradable nitinol stent. In a sheep model of CABG, this graft showed some incidence of occlusion, delamination, and aneurysmal changes by 12 months,^35^ but has advanced to a first-in-human clinical trial.

In contrast, the sdATEV does not require stenting for mechanical stability and has matrix composition and mechanical properties similar to those of native blood vessels.^21^^,^^22^^,^^24^ Thorough decellularization of ATEVs during manufacturing produces a conduit that does not induce immune response in humans: over 1,200 patient-years of exposure in nearly 600 patients receiving the 6-mm diameter ATEV have resulted in no reports of clinical immunological rejection. Non-CABG clinical experience shows that the 6-mm ATEV repopulates with vascular cells to become a living blood vessel over time;^18^ this remodeling may contribute to the observed mechanical durability of the ATEV over prolonged implantation.^20^ These prior observations showed that most of the repopulating αSMC-expressing cells are derived from progenitors in the surrounding connective tissue, which migrate into the ATEV and then differentiate into vascular smooth muscle phenotypes.^18^

The recellularization of the sdATEV that was observed in these preclinical CABG studies was similar to that observed in human explants; however, this is the first report of adaptive remodeling by transanastomotic artery medial cells to reduce diameter mismatch and WSS mismatch between the ATEV and the bypassed native blood vessels. No true neointimal hyperplasia was observed in any of the sdATEV explants. Based on histologic examination, immunohistochemistry, and spatial transcriptomics, the sdATEV neomedia comprised RCA-derived cells expressing SMC and EC markers. These cells had an extremely low proliferation rate in 3 of 5 animals: 0.35 ± 0.52% by immunostaining. In 2 of 5 animals, xenoreactive B and T lymphocytes contributed to a higher proliferative cell index in the neomedia. Despite phylogenetic similarity, the human ECM protein composition of the sdATEV is still xenogeneic to the non-immunosuppressed baboon recipients. Thus, lymphocytic infiltration and proliferation in response to the implantation of human ECM is not unexpected. Unlike human recipients of the ATEV, who do not mobilize T cells or B cells into the vessel wall after implantation,^18^ nonhuman primates have previously shown variability in immune response to the sdATEV.^21^ Therefore, the proliferating cell population in 2 of the 5 neomedia regions was likely related to infiltrating lymphocytes in this xenogeneic surgical model.

The progressive and adaptive remodeling of the sdATEV by neomedia tissue occurred predominantly between time of implantation to 3 months, and near the distal region of the sdATEV grafts, effectively reducing diameter and wall shear stress mismatch at the RCA anastomosis. Contrary to intimal hyperplasia in SVGs that forms at both anastomoses and progresses throughout the entire graft leading to occlusion within a year,^4^ the sdATEV narrowed primarily in the distal region during the first 3 months, then stabilized to quiescence by 6 months. The proximal anastomosis was created via a 3.8- to 4.0-mm aortic arteriotomy, matching the 3.5-mm sdATEV, whereas the distal anastomosis to the narrow (∼1.6-mm) baboon RCA resulted in low distal luminal shear stress due to the size mismatch. Abnormally low levels of luminal shear stress can stimulate endothelium to produce proliferative signals for SMCs, thereby thickening the wall and narrowing the lumen to restore shear stress to appropriate, “preset” levels.^36^^,^^37^ Similarly, arterial SMCs have a wall axial stress “set point,” which if exceeded will drive SMC proliferation and wall thickening until wall stress is restored to normal levels.^36^^,^^38^ Autologous veins that are used as CABG conduits experience an abrupt and permanent change in luminal shear stress and wall axial stress. These changes trigger adaptive processes, such as intimal SMC proliferation and ECM production, to return the overall state of stress to the conduit’s preimplantation homeostatic target.

The sdATEV is fundamentally different from harvested vein or artery, because it contains neither endothelium nor smooth muscle at the time of implantation, thus, no cellular injuries to the sdATEV occur during implantation. Instead, host ECs and SMCs migrate from local tissue to repopulate the sdATEV, which may facilitate adaptive remodeling within the conduit. In the native arterial wall, SMCs and ECs maintain quiescence via multiple signaling pathways, both autocrine and paracrine. Endothelial-derived nitric oxide and prostaglandins, such as PGI_2_, cause smooth muscle relaxation while also down-regulating SMC proliferation. Conversely, ET-1 causes smooth muscle constriction as well as proliferation.^39^ The human internal mammary artery endothelium has a high expression of eNOS^40^ and constitutive PTGIS^41^ with a relatively low expression of ET-1,^42^ whereas the opposite expression pattern is seen in SVGs.^43^^,^^44^ ECs lining the sdATEV at explantation had similar expression of these proteins as the endothelium of the neighboring RCA. The expression of EC proteins involved in SMC quiescence correlated with a low proliferation rate within the neomedia and expression of multiple contractile SMC markers (αSMA, CNN1, myocardin, smoothelin). Therefore, it appears that key molecules involved in homeostatic maintenance of SMC proliferation are present at similar levels in native RCA and in the sdATEV after 6 months.

### Study limitations

Given the small (n = 5 animals) cohort studied, results should be regarded as provisional. This model of a xenogeneic implantation into a non-immunosuppressed nonhuman primate limited the duration of the study to 6 months. Unfortunately, there is no established immunodeficient animal model that is large enough to study human-sized vascular conduit for CABG. In such an ideal model, longer follow-up could be achieved to further demonstrate clinical relevance. Despite being the largest primate model available, the smaller size of the baboons compared to adult humans, necessitated use of short segments of sdATEV for aorta-to-RCA bypasses compared to potential clinical application. Whereas patency, mechanical durability, and host remodeling outcomes were quite uniform among the 5 baboons, results should be corroborated in larger studies. Lastly, whereas RCA ligation mimicked total coronary occlusion, these studies were not conducted in a setting of coronary atherosclerosis.

## Conclusions

In a nonhuman primate model of CABG, the 3.5-mm sdATEV provided stable coronary blood flow and mechanical durability over a 6-month follow-up. Despite a large size mismatch at the time of implantation between the sdATEV and the native RCA, the biological nature of the sdATEV allowed for adaptive remodeling. The histologic observations at 6 months are considerably more favorable than those previously observed with synthetics, xenografts, and with allografts/cryovein. Future studies of long-term patency, durability, and vascular remodeling in an atherosclerotic model will expand understanding of the sdATEV in the human coronary circulation.

Perspectives **COMPETENCY IN MEDICAL KNOWLEDGE:** Autologous SV may not be usable in a subset of CABG patients due to a variety of reasons, which may result in incomplete revascularization. Furthermore, SV patency at 10 years after CABG remains around 50%. An off-the-shelf blood vessel bioengineered from human vascular cells would address this significant unmet need and provide cardiac surgeons with an alternative, readily available conduit for CABG. More importantly, remodeling of the sdATEV by host coronary artery cells already attuned to the local environment regulate blood flow and may result in superior long-term patency than harvested SV.

**TRANSLATIONAL OUTLOOK:** These results from a non-immunosuppressed large primate model of CABG demonstrate no evidence of graft thrombosis, dilatation, or significant stenosis through 6 months. Indeed, these findings support further evaluation of the sdATEV in first-in-man clinical trials in patients undergoing CABG.

## Funding Support and Author Disclosures

The work described in this paper was fully funded by Humacyte Global, Inc. Drs Nash, Kirkton, Levitan, Naegeli, Prichard, and Niklason are employed by Humacyte Global, Inc and own stock or stock options in Humacyte Global, Inc. Drs Benkert and McCartney, have received consulting fees from Humacyte Global, Inc. Dr Kypson has received consulting fees from Humacyte Global, Inc.; and owns stock or stock options in Humacyte Global, Inc. All other authors have reported that they have no relationships relevant to the contents of this paper to disclose.

## Appendix

Supplemental Figures 1-5 and Supplemental Tables 1-4

Video 1 Post-operative video of the sdATEV as aorta-to-RCA conduit on the baboon heart.

Video 2 Fluoroscopic angiography of sdATEV aorta-to-RCA conduit 10 days after implantation.

Video 3 Fluoroscopic angiography of sdATEV aorta-to-RCA conduit 6 months after implantation.

Video 4 End of study explant video of sdATEV aorta-to-RCA conduit 6 months after implantation.
