# Supplementary material for: Acellular Tissue Engineered Vessels as Coronary Artery Bypass Grafts
Source: JACC Basic Transl Sci. 2025 Sep 11;10(10):101379. doi: 10.1016/j.jacbts.2025.101379 (PMC12665417; doi:10.1016/j.jacbts.2025.101379)
Supplement: Supplemental Figures 1-5 and Supplemental Tables 1-4 [file mmc1.docx]

**Supplemental Appendix**

Acellular Tissue Engineered Vessels as Coronary Artery Bypass Grafts

**Authors:** Adam R. Williams MD^a,f^, Kevin M. Nash PhD, DABT^b,f^, Robert D. Kirkton PhD^b^, Garyn S. Levitan BS^b^, Melissa A. Daubert MD, FASE, FACC, FSSCT^c^, Susan A. Whitney MBA, BSRT(R)(N)(CT)^c^, Kaleb M. Naegeli PhD^b^, Abigail R. Benkert MD^a^, Sharon L. McCartney MD^d^, Heather L. Prichard PhD^b^, Laura E. Niklason MD, PhD^b^, and Alan P. Kypson MD^b,e,*^

**Affiliations:**

^a^Duke University Medical Center, Department of Surgery; Durham, NC, USA

^b^Humacyte Global, Inc; Durham, NC, USA

^c^Duke University Medical Center, Department of Medicine and Radiology; Durham, NC, USA

^d^Duke University Medical Center, Department of Anesthesiology; Durham, NC, USA

^e^University of North Carolina Healthcare, REX Cardiac Surgical Specialists; Raleigh, NC, USA

^f^These authors contributed equally

*Corresponding author. Email: alan.kypson@unchealth.unc.edu

This file includes:

Supplemental Tables 1-4

Supplemental Figures 1-5

| **Supplemental Table 1. sdATEV baboon CABG summary** | | | | | | | | |
| --- | --- | --- | --- | --- | --- | --- | --- | --- |
| **Animal** | **Age (yr)** | **Weight (kg)** | **sdATEV Length (cm)** | **Implant Duration (days)** | **Day 0 TTFM (mL/min)** | **Day 0  PI** | **Month 6 TTFM (mL/min)** | **Month 6  PI** |
| 31778 | 9 | 31.5 | 3.2 | 190 | 34 | 1.7 | 28 | 1.2 |
| 31506 | 10 | 30.5 | 3.5 | 162 | 19 | 2.3 | 21 | 1.2 |
| 32582 | 8 | 37.0 | 2.0 | 175 | 25 | 0.8 | 38 | 1.2 |
| 36285 | 6 | 34.8 | 2.5 | 182 | 42 | 0.8 | 52 | 1.2 |
| 35139 | 8 | 32.5 | 2.5 | 189 | 42 | 2.9 | 36 | 1.1 |
|  |  |  | **Average (±SD):** | | 32 (±10) | 1.7 (±0.9) | 35 (±13) | 1.2 (±0.0) |
| sdATEV: small diameter acellular tissue engineered vessel; PI: pulsatility index; TTFM: transit time flowmetry | | | | | | | | |

| **Supplemental Table 2. Antibodies used for immunohistochemistry** | | | | |
| --- | --- | --- | --- | --- |
| **Antibody:**​ | **Host Species:**​ | **Company:**​ | **Cat.#**​ | **Dilution / Concentration Used:**​ |
| αSMA​ | mouse​ | Dako​ | M0851​ | 1:200​ |
| αSMA​ | rabbit​ | Abcam​ | ab5694​ | 1:200​ |
| CD3​ | rabbit​ | Dako​ | A0452​ | 1:200​ |
| CD20​ | mouse​ | Abcam | ab9475 | 1:200​ |
| CD31​ | mouse​ | Dako​ | M0823​ | 1:200​ |
| CNN1​ | mouse​ | Abcam​ | ab700​ | 1:200​ |
| eNOS​ | mouse​ | BD​ | ​610297 | 1:50​ |
| ET-1 | mouse | Invitrogen | MA3-005 | 1:250 |
| Ki67 | mouse | Cell Signaling Technologies | 9449 | 1:200 |
| Myocardin | mouse | R&D Systems | MAB4028 | 1:200 |
| PTGIS | rabbit | Abcam | ab23668 | 4 µg/mL |
| Smoothelin | mouse | EMD Millipore | MAB3242 | 1:100 |
| TGFβ1 | mouse | Thermo Fisher | MA1-21595 | 1:100 |
| vWF | rabbit | Abcam​ | ab179451 | 1:250 |
| DAPI | N/A | Thermo Fisher | 62248 | 1:1000 |
| goat anti-mouse IgG Alexa Fluor 488​ | goat​ | Thermo Fisher​ | A-11001​ | 1:400​ |
| goat anti-rabbit IgG Alexa Fluor 594​ | goat​ | Thermo Fisher​ | A-11012​ | 1:400​ |

**Supplemental Table 3. Spatial transcriptomics—Top 10 genes distinguishing RCA layers and their expression in sdATEV neomedia**


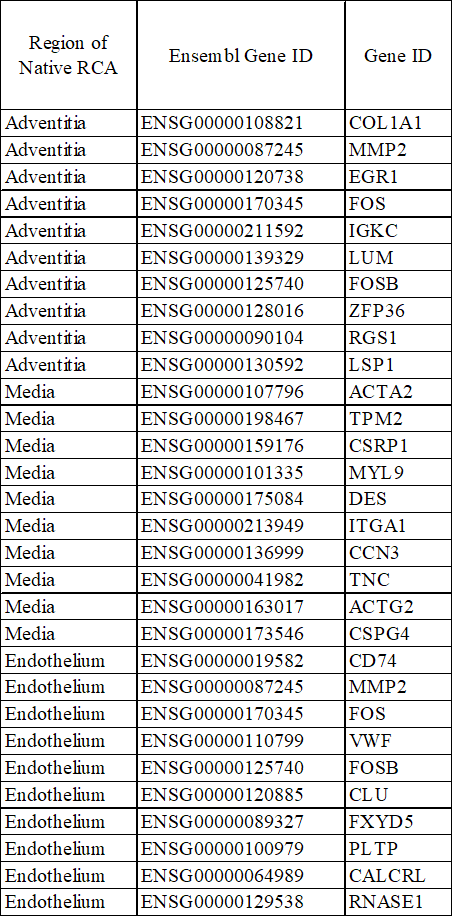


**Supplemental Table 4. Spatial transcriptomics—Gene families utilized in Figure 6 and Supplemental Figure 5**

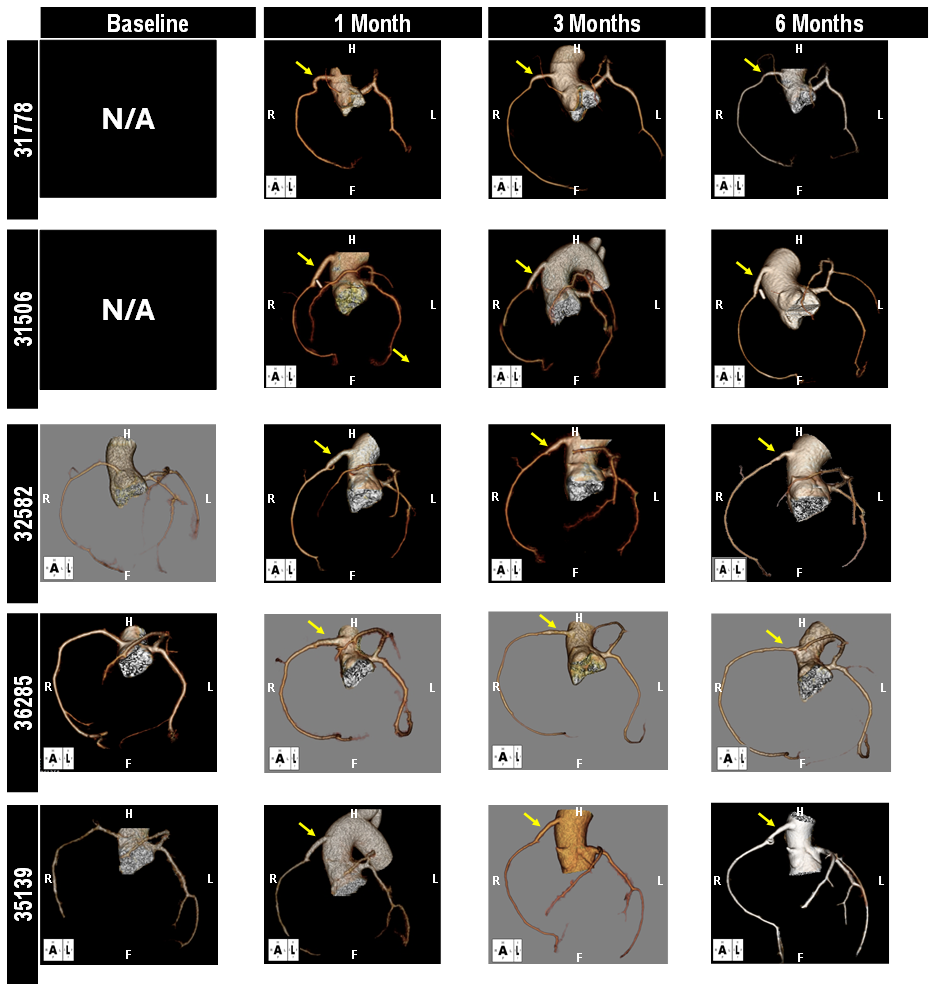


**Supplemental Figure 1.** CTA for all study animals at pre-operative baseline, 1 month, 3 months, and 6 months after implantation. Baseline CTA not available (“N/A”) as it was not performed on first two animals. Yellow arrows denote location of the sdATEV conduit.


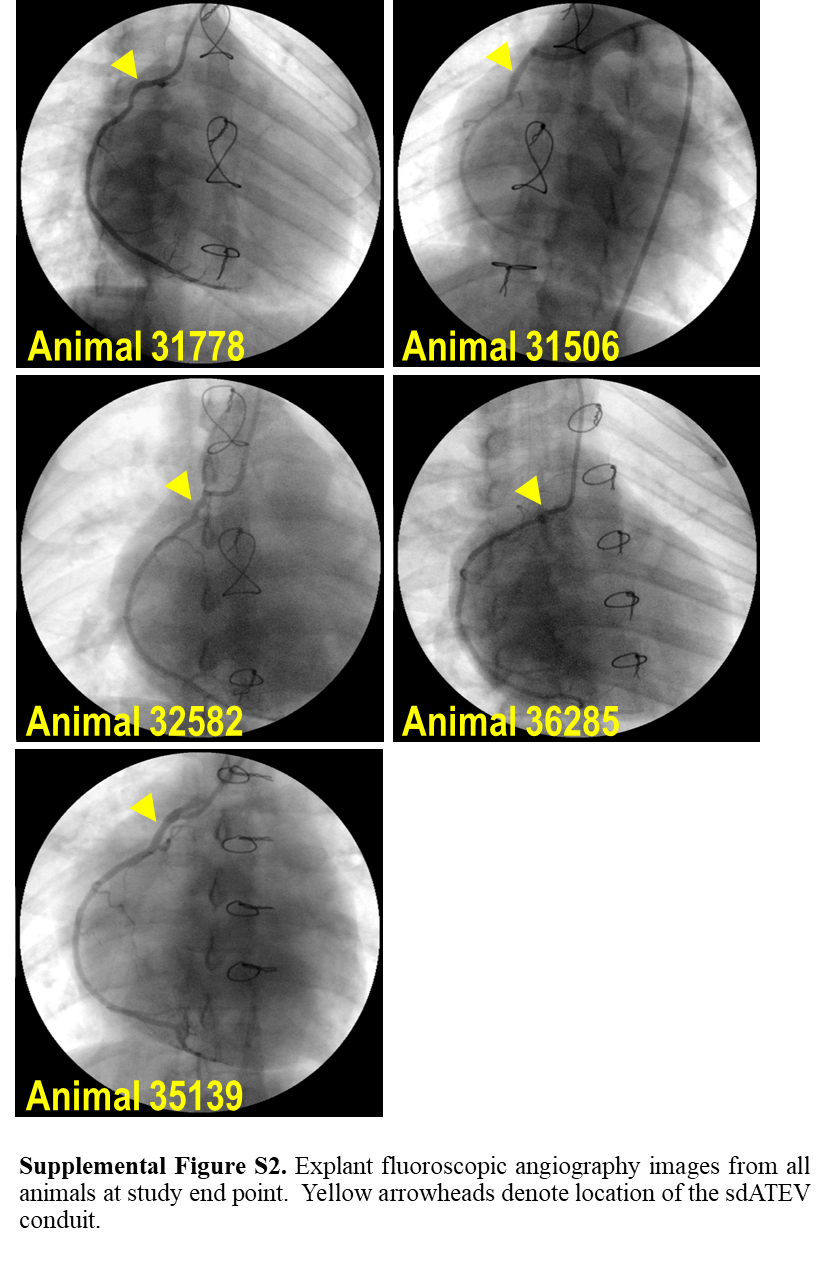


**Supplemental Figure 2.** Explant fluoroscopic angiography images from all animals at study endpoint. Yellow arrowheads denote location of the sdATEV conduit.


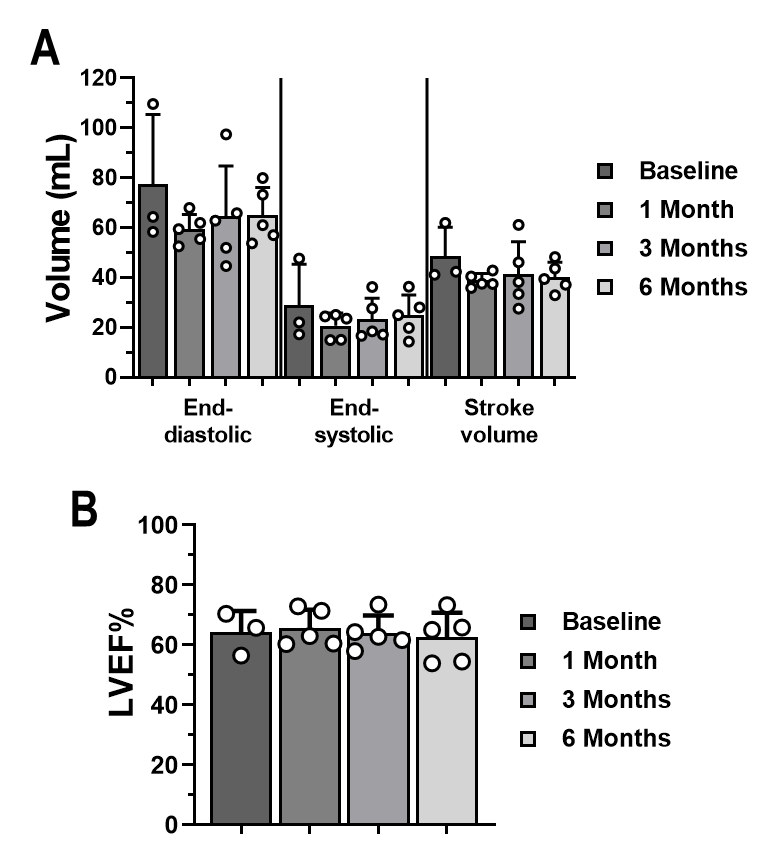


**Supplemental Figure 3. Heart function of animals during 6 month study.** (A) End-diastolic, end-systolic, and stroke volumes for 5 animals at baseline, 1, 3 and 6 months. Circles indicate data from each animal with no significant trends observed. (B) Left ventricular ejection fraction (LVEF) is unchanged through 6 months. Data represented as means ± SD; individual datapoints depicted as open circles; one-way ANOVA, followed by Tukey’s multiple comparisons test.


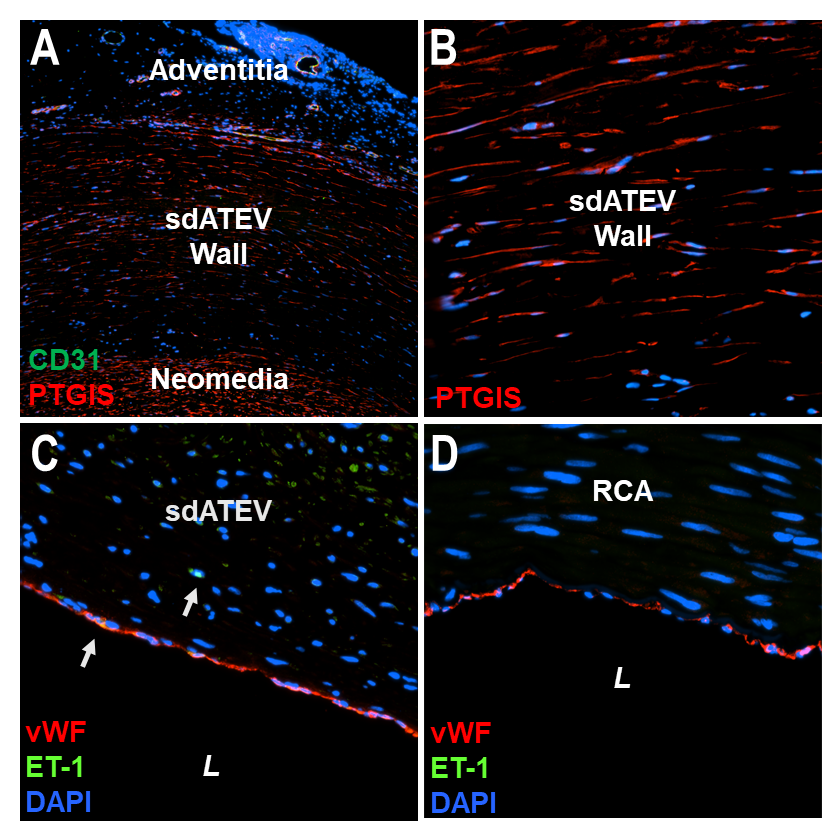


**Supplemental Figure 4. Representative immunohistochemical staining of PTGIS and ET-1 in sdATEV and RCA 6-month explants.** (A, B) Prostacyclin PGI_2_ synthase (PTGIS) weakly expressed (red) in many CD31^-^ cells within the sdATEV neomedia and wall. (C, D) Endothelial cells stained positively for vWF (red) rarely co-expressed (white arrows) endothelin-1 (ET-1, green) on the lumen of sdATEV explants (C) consistent with lack of ET-1 expression in luminal cells on the RCA (D).


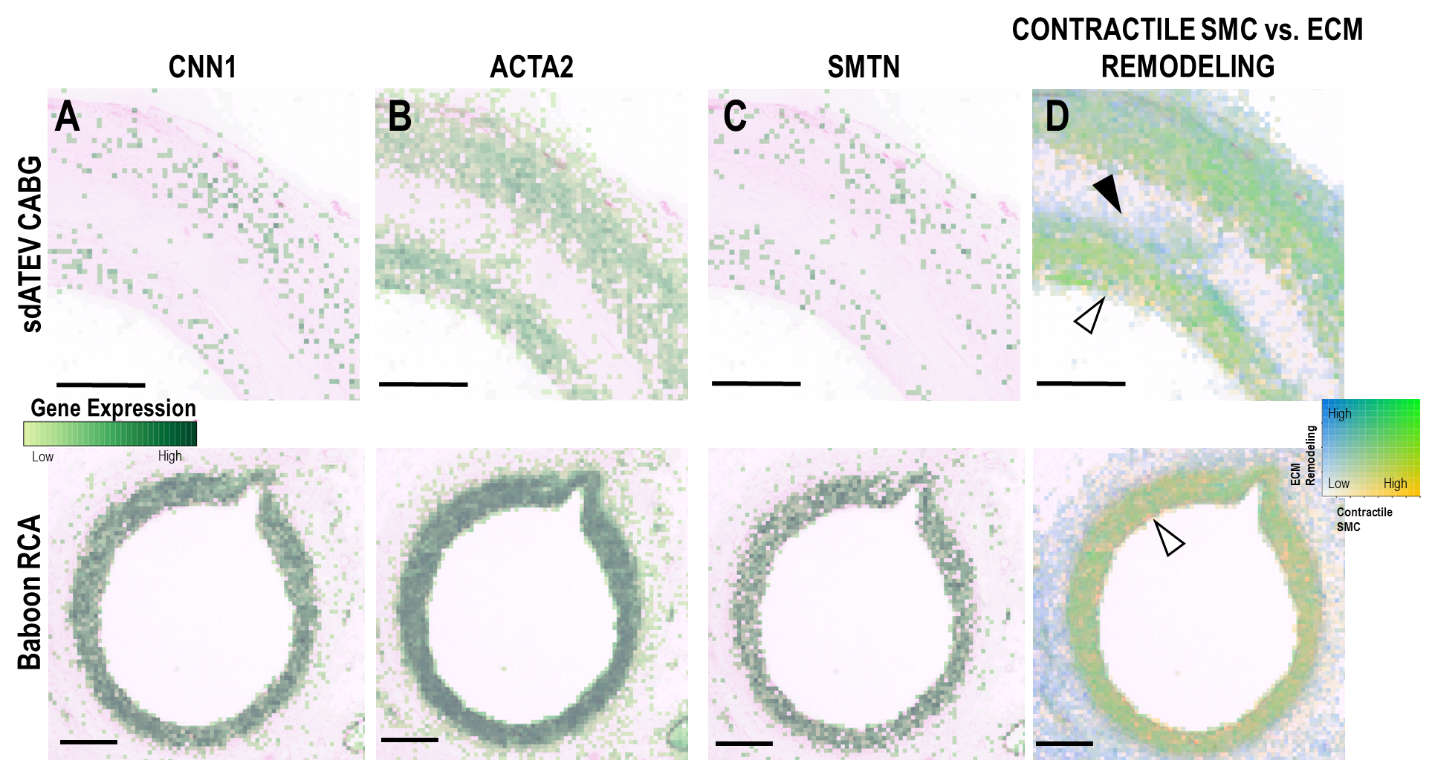


**Supplemental Figure 5. Expression of Contractile Smooth Muscle Markers in sdATEV CABG Explant.** Spatial transcriptomic gene expression patterns for the contractile smooth muscle markers calponin (CNN1, A), smooth muscle actin (ACTA2, B), and smoothelin (SMTN, C) in a midgraft tissue section of the explanted sdATEV (top row) and native RCA (bottom) from same animal. (D) Co-expression of selected contractile smooth muscle markers (ACTA2, CNN1, MYH1, MYOCD, SMTN, and TAGLN) compared with expression of ECM remodeling genes (AEBP1, COL11A1, COL5A1, COL6A2, LOX, POSTN, SNAI2, THBS2, TIMP3, VCAN) demonstrates a layer of advancing ECM reconstruction (black, arrowhead) ahead of the contractile smooth muscle (green) and co-expressing cells (yellow) in the neomedia and artery wall of the RCA (white arrowheads). Scale bars = 500 μm
